# Proteinaceous Nano container Encapsulate Polycyclic Aromatic Hydrocarbons

**DOI:** 10.1038/s41598-018-37323-x

**Published:** 2019-01-31

**Authors:** Matthew McDougall, Olga Francisco, Candice Harder-Viddal, Roy Roshko, Fabian Heide, Shubleen Sidhu, Mazdak Khajehpour, Jennifer Leslie, Vince Palace, Gregg T. Tomy, Jörg Stetefeld

**Affiliations:** 1 Department of Chemistry, University of Manitoba, 144 Dysart Road, Winnipeg, Manitoba R3T 2N2 Canada; 2 Centre for Oil and Gas Research and Development (COGRAD), 144 Dysart Road, Winnipeg, MB R3T 2N2 Canada; 3 Department of Chemistry and Physics, Canadian Mennonite University, 500 Shaftesbury Blvd, Winnipeg, MB R3T 2N2 Canada; 4 Department of Physics and Astronomy, University of Manitoba, 30A Sifton Rd, Winnipeg, MB R3T 2N2 Canada; 5 Stantec Consulting Inc., 500-311 Portage Ave., Winnipeg, MB R3B 2B9 Canada; 6 IISD-Experimental Lakes Area, 111 Lombard Ave, Winnipeg, MB R3B 0T4 Canada

## Abstract

Polycyclic aromatic hydrocarbons (PAHs) are toxic, mutagenic and among the most damaging chemical compounds with regard to living organisms. Because of their persistence and wide distribution removal from the environment is an important challenge. Here we report a new Nano container matrix based on the deep sea archaea-derived RHCC-Nanotube (RHCC-NT), which rapidly and preferentially binds low molecular weight PAHs. Under controlled-laboratory conditions and using fluorescence spectroscopy in combination with X-ray crystallography and MD simulations, we quantified the real-time binding of low molecular weight PAHs (2–4 rings) to our substrate. Binding coefficients ranged from 5.4 ± 1.6 (fluorene) to 32 ± 7.0 μM (acenaphthylene) and a binding capacity of 85 pmoles PAH per mg RHCC-NT, or 2.12 μmoles in a standard 25 mg sampler. The uptake rate of pyrene was calculated to be 1.59 nmol/hr∙mol RHCC-NT (at 10  C). Our results clearly show that RHCC-NT is uniquely suited as a monitoring matrix for low molecular weight PAHs.

## Introduction

PAHs are a class of organic compounds that consist of two ore more fused benzene rings forming ring-shaped super molecules. PAHs are important drivers of chronic toxicity to aquatic organisms^1,2^. They are extremely restive molecules that can persist in the environment due their hydrophobicity and low water solubility^3,4^. As a result, they are ubiquitous in air, water and soil at varying concentrations depending on proximity to point and non-point sources^1^. While they are generally lipophilic, some PAHs are more soluble in water and are highly toxic to aquatic biota, including fish and invertebrates^5^. Specifically, PAHs with 3–5 aromatic rings are the most important source of chronic toxicity to aquatic organisms exposed at e.g. oil spill sites^6,7^. Because of the wide distribution of PAHs, it is important to understand natural and anthropogenic sources of these compounds and to monitor their baseline concentrations in the aquatic environment^8^.

Discovered growing on the periphery of deep-sea hydrothermal vents, *Staphylothermus marinus* is a heterotrophic hyperthermophilic archaea that requires elemental sulfur for growth. The ability of *S. marinus* to sequester elemental sulfur from its environment relies in part on the Right Hand Coiled-Coil Nanotube (RHCC-NT), a protein fragment in the surface layer of the microorganism. The RHCC-NT encapsulates elemental cyclo-octasulfur in a hydrophobic cavity with a diameter of ~8.4 Å, where it is then available for metabolism. These cavities, adapted to capture the small, hydrophobic cyclo-octasulfur are also of a suitable size to encapsulate low molecular weight PAHs^9^. Due to the extreme conditions of its origin the RHCC-NT can function with a wide range of temperature and chemical stability, and would easily maintain its fold and binding capacity in aquatic environments surrounding hydrocarbon extraction and transportation sites.

**Figure 1:**
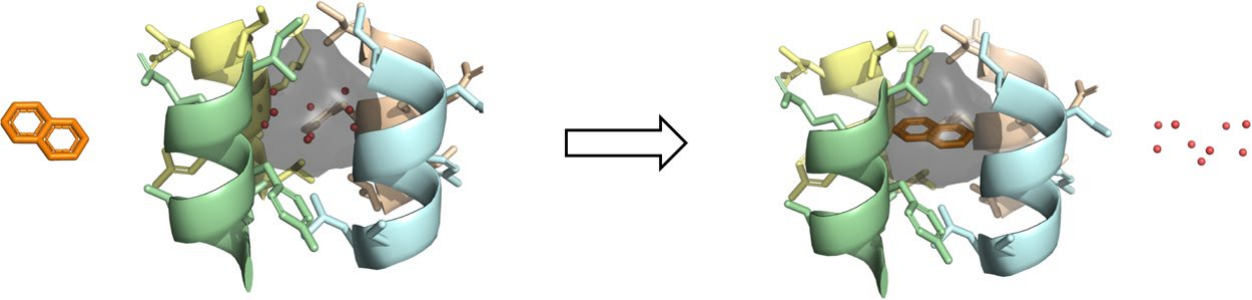
Upon binding the RHCC-NT, the PAH displaces the waters in the cavity and moves from an aqueous environment to the hydrophobic interior of the protein. Due to the fluorescent hydrophobic effect, this change in environment results in an increase in quantum yield, a shift in the emission maximum, or both.

Here we present an in-depth characterization of RHCC-NT and its unique uptake properties for PAHs (Fig. 1). Specifically, we quantified the capacity of RHCC-NT to bind 7 lower MW weight PAHs using their intrinsic fluorescence in steady-state binding experiments. We also studied the ability of the nanotube to uptake PAHs by using pyrene as a model compound. Moreover, these binding experiments were coupled with calculations of the transfer free energy based on molecular dynamics simulations and structural studies based on X-ray crystallography to determine the PAH location within the RHCC-NT. Our studies provide an insight for future binding site optimization within the nanotube, with the end goal to use it as a monitoring device in the field application.

## Results

### Fluorescence monitoring studies and binding constants

**Figure 2:**
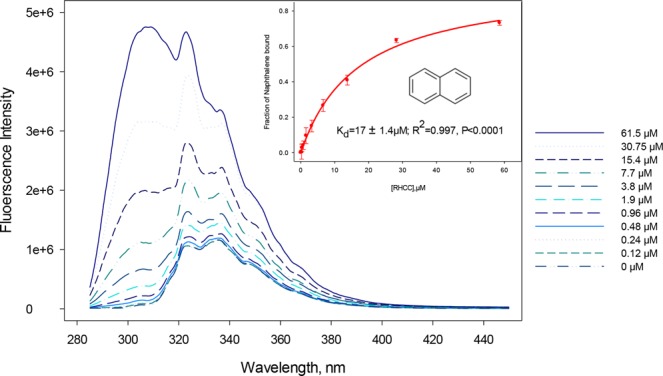
Fluorescence emission spectra (λ_exc_ = 275 nm) of 4 µM Naphthalene at different RHCC-NT concentrations. A maximum was assigned at 350 nm to eliminate any contribution from tyrosine fluorescence present in RHCC-NT. Slits were set to 1 nm. The inset shows the fraction of Naphthalene bound plotted as a function of RHCC-NT concentration.

**Figure 3:**
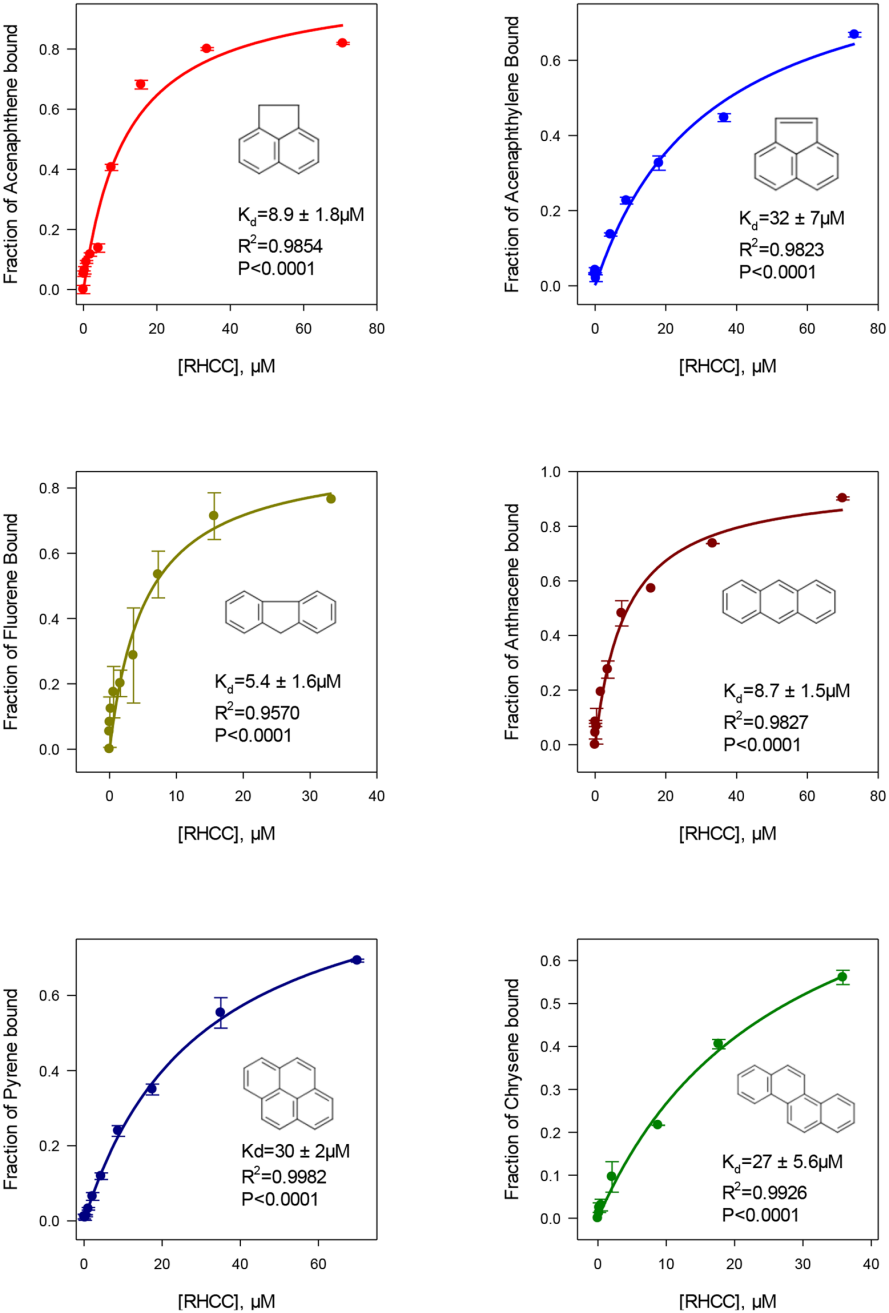
Binding capacity of RHCC-NT in uptake of lower MW PAHs in aqueous solution. PAH binding constants were calculated based on the Equation (1) using the same principle described in Methods section. Fluorescence emission maxima were assigned as following: acenaphthene 322 nm, acenaphthylene 323 nm, fluorene 315 nm, anthracene 421 nm, pyrene 384 nm, chrysene 400 nm.

Fluorescence assays were used to examine the binding of RHCC-NT to PAHs as they allow discrimination between PAHs in solution and those bound to the RHCC-NT, as well as the ability to monitor PAH uptake as a function of time (Fig. 2). This is possible due to the polarity effect exhibited by many fluorophores, where changes in the polarity of the immediate environment of the fluorophore results in changes in the intensity or peak position in the fluorescence spectrum; the capacity of RHCC-NT to bind PAHs is examined by the changes in the PAH fluorescence spectrum as a function of increasing nanotube concentrations. Titration of RHCC-NT into a naphthalene solution results in an increase of its quantum yield consistent with the movement of naphthalene into a lower polarity environment. These fluorescence data could then be fit to Equation (1), yielding a binding constant for naphthalene of *K*_*d*_ = 17 ± 1.4 µM (Fig. 2). It must be noted that the RHCC-NT also fluoresces when excited at 275 nm (Figure S1); therefore, it was necessary to correct for this contribution in the binding analysis. In order to minimize contributions from tyrosine fluorescence, the fluorescence emission at 350 nm was used to determine the binding affinity of naphthalene to RHCC-NT (Table 1). The approach described above was applied to the analysis of other low molecular weight PAH’s fluorescence data (Fig. 3), however, excitation wavelengths longer than 275 nm were available for the remaining PAHs and RHCC-NT interference was minimized (for excitation wavelengths refer to Methods).

**Table 1:** Change in measured fluorescence intensity of naphthalene at 350 nm with increasing RHCC concentration.

[RHCC], µM	Fluorescence intensity at 350 nm (λ_exc_ = 275 nm)
0	708292
0.12	709824
0.24	730572
0.48	733694
0.96	780644
1.9	906120
3.8	1003650
7.7	1177910
15.4	1420260
30.75	1845910
61.5	1873130

Though phenanthrene, fluoranthene, and benz(a)anthracene were also tested (Figure S2), they did not exhibit any concentration dependent fluorescence changes. The maximum dimension of these compounds and their hydrophobicity are not significantly different from the other low MW PAHs examined, however, they do exhibit a lower degree of symmetry. In general, electron distributions in the π orbitals have a significant effect on the affinity of the RHCC-NT for structurally similar PAHs. Whereas acenaphthene yields a *K*_*d*_-value of 8.9 ± 1.8 µM, acenaphthylene is bound significantly weaker (*K*_*d*_ = 32 ± 7.0 µM) (Table 2). Naphthalene, of particular interest with respect to toxicity, binds with a *K*_*d*_ of 17 ± 1.4 µM. These results suggest that RHCC will be most useful as a passive sampling media, for binding symmetrical 2–4 ring symmetrical PAHs. Introducing alkyl groups to the structure of the parent PAHs will impact symmetry and lipophilicity^10^, which may also impact binding to the RHCC matrix.

**Table 2:** *K*_*d*_ values and molar Gibbs Free energy for individual PAHs

Compound	*K*_*d*_, µM	R^2^-value	∆G, kJ/mol^a^
Fluorene	5.4 ± 1.6	0.9570	−29.9
Anthracene	8.7 ± 1.5	0.9827	−28.7
Acenaphthene	8.9 ± 1.8	0.9854	−28.6
Naphthalene	17 ± 1.4	0.9970	−27.1
Chrysene	27 ± 5.6	0.9926	−25.9
Pyrene	30 ± 2.0	0.9982	−25.6
Acenaphthylene	32 ± 7.0	0.9823	−25.5

^a^Molar Gibbs free energy for the ligand binding is defined as ∆G = RT ln (*K*_*d*_).

### Time-scale study

**Figure 4:**
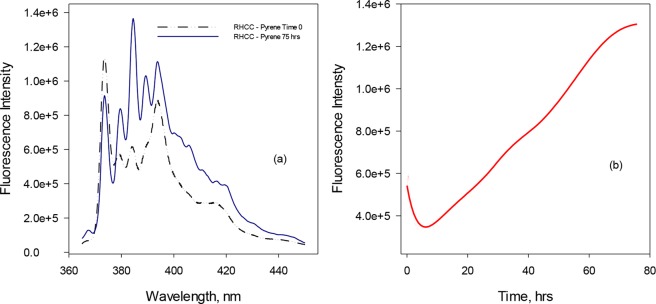
Time-scale uptake of pyrene by RHCC-NT. (**a**) Fluorescence emission spectrum (λ_exc_ = 334 nm) of 2 µM pyrene measured in the presence of 70 µM RHCC at the beginning of experiment (dashed line -Time 0) and after 75 hours (solid line) when reaction reached complete equilibrium. A maximum was assigned at 384 nm. Slits were set to 1 nm. (**b**) Fluorescence emission maxima at 384 nm measured during the course of the experiment plotted as a function of time to show time-dependent uptake of pyrene by RHCC-NT.

To probe the uptake kinetics of low MW PAHs, we examined the fluorescence characteristics of pyrene in solvent in comparison to RHCC-NT bound pyrene (Fig. 4). As the bulkiest of the PAHs tested, it is expected that pyrene provides an upper bound on the equilibration time of these low MW PAHs. We took advantage of the major vibronic bands (I and III) of pyrene with defined peaks at ~373 and 384 nm^11,12^. In comparison to the emission intensity of Band I at 373 nm, the intensity of Band III at 384 nm is significantly enhanced in hydrophobic environments. In contrast, the intensity of Band I is significantly higher than that of Band III in polar environments. The ratio of the fluorescence emission intensities of Band I/III is defined as *Py*-value^13,14^ and can be employed to detect polarity in the vicinity of the probed microenvironment. Our findings clearly demonstrate a *Py* shift from 1.7288 (RHCC-NT:Pyrene time = 0: (I_I_/I_III_ = 1067230/617334) to *Py* = 0.6395 (RHCC-NT:Pyrene time = 75 hrs: (I_I_/I_III_ = 834882/1305448). Figure 4 demonstrates the change in pyrene fluorescence emission spectrum with the change of the polarity. A significant increase in the intensity at 384 nm (Band III) is observed when pyrene is incorporated into the hydrophobic microenvironment of the RHCC-NT cavities. By monitoring the change in pyrene emission at 384 nm and plotting this change as a function of time we determined that complete uptake of pyrene by RHCC-NT at 10 °C takes around 80hrs, with an average uptake rate of 1.59 nmol/hr∙mol RHCC-NT. Under these conditions, we found that naphthalene, acenaphthene and acenaphthylene all reach equilibrium in *ca*. 1 min (data not shown).

### Structural elucidation of RHCC-NT in complex with naphthalene and pyrene

**Figure 5:**
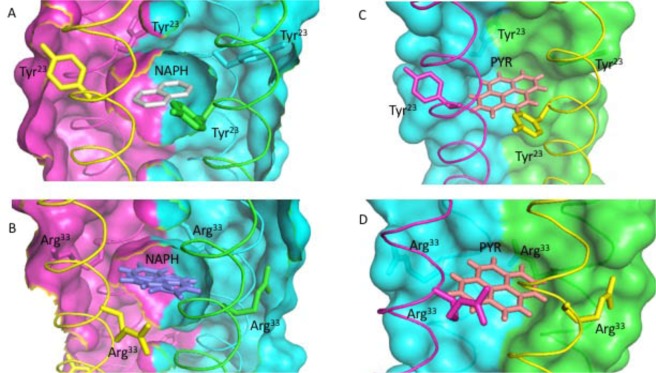
X-ray Crystal structures of Naphthalene (NAPH; **A**,**B**) and Pyrene (PYR; **C**,**D**). Cross sections of cavities 2 (**A,C**) and 3 (**B,D**) are drawn with individual chains of the tetrameric RHCC-NT shown in different colors. To mark cavities 2 and 3, tyrosine moieties of Tyr^23^ and the guanidine moieties of Arg^33^ are highlighted in stick mode.

To verify our previous findings and to explore the molecular mechanisms of PAH uptake we determined the X-ray crystal structures of RHCC-NT in complex with both naphthalene and pyrene (Fig. 5 and Table 3). Our findings reveal that the RHCC-NT binds both 2- and 4- ring PAHs in the same hydrophobic pockets, justifying the use of a common model to measure the binding coefficients through the subset of PAHs examined in this study. Comparing unliganded *vs* liganded structures, there is no major conformational rearrangement at the protein backbone nor of individual side chains detectable. The large-sized cavities act as fixed size container, which are able to uptake large hydrophobic ring-like structures. Remarkably, in the unliganded RHCC-NT structures, the cavities are filled with water clusters (pdb:1FE6)^15^. As these cavities are hydrophobic in nature, it is expected that the presence of polar waters in the cavities is unfavorable relative to a less hydrophilic moiety. Indeed, both naphthalene and pyrene completely displace the water molecules within the binding pocket (Figure S4). The plane of the PAHs lies perpendicular to the long axis of the RHCC-NT, even though the cavity is slightly elongated along this helical axis. This could be due to the interaction of the π- electron cloud with the dipole of the nanotube, though further experimentation will be necessary to confirm this.

**Table 3:** X-ray structure determination.

Data Collection	RHCC-Naphthalene (5VKF)	RHCC-Pyrene (5VH0)
λ (Å)	1.54192	1.54192
Space Group	P31 2 1	P1 2 1
**Cell dimensions**		
*a, b, c* (Å)	110.36 110.36 70.83	35.02 77.00 35.10
*α, β, γ* (°)	90.00 90.00 120.00	90.00 90.00 90.00
No. reflections	66293 (9151)	42470 (2720)
Resolution (Å)	19.12–2.75 (2.90–2.75)	19.25–2.06 (2.11–2.06)
*R*_*merge*_	0.167 (0.689)	0.157 (0.789)
*I/*σI	7.2 (2.4)	8.3 (2.4)
Completeness (%)	98.9 (95.6)	99.3 (93.4)
Multiplicity	5.1 (5.0)	3.7 (3.3)
**Refinement**		
*R*_*work*_ */R*_*free*_^b^	0.199/0.239	0.212/0.259
**No. atoms**		
Protein	1689	1510
Ligand/Ion	45	68
Water	126	109
*B-*factor (Å^2^)		
Protein	42.32	35.57
Water	35.51	35.42
Ligands	74.53	40.31
**R.m.s. deviations**		
Bond lengths (Å)	0.002	0.003
Bond angles (°)	0.33	0.41

^a^Statistics of the highest resolution shell are shown in parenthesis. ^b^The R_free_ was calculated by selecting 10% of observed reflections from refinement.

### Molecular Dynamics Simulations

The computed values of the transfer free energy for the three PAH ligands are listed in Table 4 for both cavities 2 and 3. The calculated solvation free energies ${\rm{\Delta }}{G}_{solv}=-{\rm{\Delta }}{G}_{3}$ in Table 4 are comparable in magnitude to the measured solvation free energies^16^, Δ*G*_*solv*_(*naphthalene*) = −10.0 ± 2.5,Δ*G*_*solv*_(*phenanthrene*) = −16.2 ± 2.5, Δ*G*_*solv*_(*pyrene*) = −18.91 ± 2.5 and show the same systematic trend through the series naphthalene → phenanthrene → pyrene. The calculated transfer free energies ${\rm{\Delta }}{G}_{transfer}^{0}$ for naphthalene and pyrene in Table 4 are significantly larger in magnitude than the measured values in Table 2. Such systematic discrepancies between calculated and measured binding free energies have been reported elsewhere in the literature when MCTI is used to compute *absolute* binding energies in strongly hydrophobic systems. In particular, the absolute binding free energy of isobutyl-methoxy-pyrazine (IMBP) bound to porcine odorant binding proteins (OBP) is computed to be ~−67 *kJ*/*mol*^17^ compared to the experimental value of −38.5 *kJ*/*mol* (similar to the discrepancy in the current investigation) and the absolute binding free energy of FKBP, a protein from the immunophilin group, bound to several ligands has been shown to be systematically more negative than the experimental values by ~13.4 *kJ*/*mol*^18^. By contrast, the application of MCTI to more hydrophilic systems yields much better agreement. This behaviour appears to be independent of the choice of force field, sampling time or implicit versus explicit solvent models and has been attributed to the higher mobility of ligands in hydrophobic cavities although, thus far, no successful protocols have emerged for improving the discrepancy. Nevertheless, the calculated binding free energies do correctly reproduce the experimental systematics with $|{\rm{\Delta }}{G}_{transfer}^{0}(naphthalene)| > |{\rm{\Delta }}{G}_{transfer}^{0}(pyrene)|$.

**Table 4:** Contributions to the free energy of transfer (kJ mol^−1^) from MD simulations.

PAH	cavity	∆G_1_	∆G_2_	∆G_3_	${\rm{\Delta }}{{\bf{G}}}_{{\bf{transfer}}}^{0}$
naphthalene	2	79.2 ± 1.2	−1.5	4.9 ± 1.0	−72.8 ± 1.1
	3	74.7 ± 1.4	−1.5	4.9 ± 1.0	−68.3 ± 1.2
phenanthrene	2	80.2 ± 3.8	−1.5	10.1 ± 1.1	−68.6 ± 2.8
	3	58.7 ± 3.8	−1.5	10.1 ± 1.1	−47.1 ± 2.8
pyrene	2	76.3 ± 8.8	−1.5	12.5 ± 1.1	−62.3 ± 6.3
	3	62.7 ± 2.9	−1.5	12.5 ± 1.1	−48.7 ± 2.2

The MD simulations predict that phenanthrene is also stable in both cavities, in spite of its lower degree of symmetry, with a transfer free energy intermediate between that for naphthalene and pyrene in cavity 2 and equal to that for pyrene in cavity 3. The discrepancy between MD simulations and experiment in the case of phenanthrene is most likely the result of a limitation on the fluorescence technique. Other techniques (GC-MS/MS) clearly show an uptake of all 2, 3 and 4 ring PAHs by RHCC-tetrabrachion, including phenanthrene, fluoranthrene and benz(a)anthracene.

A visual examination of the fully interacting, *FBHW* − restrained, *λ* = 0 trajectories frame-by-frame showed that, on average, the plane of all three PAH ligands maintained an orientation transverse to the central cavity of the RHCC-NT throughout the entire trajectory, as observed experimentally, with angular fluctuations Δ*θ* ~± 10  relative to the initial orientation. However, all three PAH ligands exhibited rapid transitions between discrete orientations within the transverse plane, with an angular separation of Δ*ϕ*~30 .

## Discussion

Using fluorescence probe techniques, X-ray crystallography, and Molecular Dynamics Simulations we have demonstrated that the RHCC-NT can bind low MW PAHs with low equilibration times and high selectivity. To our knowledge, this is the first study showing that a proteinaceous Nano container can encapsulate PAHs. The cavities of RHCC-NT are ellipsoidal in shape with a volume of ~380 Å^3^ and allow for the uptake of PAHs up to 4-rings in size, while binding was not detected for any 5 or higher ring PAH systems. This suggests that such molecules may be too large to fit inside the cavities.

Coiled coils are mostly known as strict oligomerisation motifs which function as entropic clamps to oligomerise target systems and to increase the local copy number of protein domains^19,20^. However, most recent studies have shown that RHCC-NT and COMPcc can uptake a number of different cargos, including vitamin D3^21^, fatty acids^22^, S8 crowns^9^ and metallic Hg cluster^23^. Whereas COMPcc shows a very dynamic behavior and extensive “breathing effect” during uptake of the ligand via the open N-terminal chamber, the RHCC-NT is a highly rigid homo-tetramer, which seems to allow uptake of hydrophobic cargos just via the inter-helical space. The closed storage cavities of RHCC-NT are very rigid and do not show any sign of significant structural re-arrangements upon binding. A common feature for both systems is that strictly hydrophobic interior channels are releasing water clusters upon hydrophobic ligand binding.

The genotoxoicity of PAHs is linked to metabolic activation via cytochrome P450 family enzymes into highly reactive electrophiles interacting with DNA sites^24^. The crystal structure of human P450 1A2 reveals a compact, highly adaptive active site pocket for positioning and oxidation of the relatively large planar disk-shaped structures^25^. In general, these metabolites are considerably more toxic than the PAH precursor molecules. There is a wealth of studies published describing the bioremediation of PAHs by pollutant-degrading microorganisms^26^. However, many environmental factors (incl. pH, Temperature, salinity, nutrient availability and bioavailability of the contaminant) play a crucial role in these processes and are difficult to control.

In conclusion, we have successfully studied the unique encapsulation of low MW PAHs in Nano containers of the archaea RHCC-NT. As shown in a most recent study, the large-sized cavities of RHCC-NT are not only able to store elemental sulfur S8 crowns^9^, however the Nano container can also reduce and store metallic metal clusters^23^. Moreover, RHCC-NT can be seen as a new protein-based matrix for removing PAHs and heavy metals from water. Using fluorescence probe techniques and X-ray crystallography we have demonstrated that RHCC-NT provides many advantages over traditional Passive Sample Devices for low MW PAHs. With low equilibration times and selective binding, the biodegradable RHCC-NT is ideally suited for determining changes in PAH concentrations at an oil spill site. This is critical as proportions of the toxic 3–5 ring PAHs changes rapidly at a spill site due to weathering and traditional PAH PSD devices take too long to reach equilibrium for any meaningful short-term measurements. In addition, our RHCC-NT matrix is extremely robust and because it is not susceptible to chemical changes because of temperature and/or pH it has the potential to be used in a wide range of aquatic environments. Further work is ongoing in our group to evaluate the performance of the RHCC-NT in the field.

## Methods

### Chemicals Reagents

Individual PAHs (naphthalene, acenaphthene, anthracene, pyrene, chrysene, fluorine and acenaphthylene) were purchased from AccuStandard (New Haven, CT) as 1000 µg/µL solutions in methanol. Ethanol (99.8% HPLC grade), sodium chloride (CAS No. 7647-14-5), sodium dihydrogen phosphate monohydrate (CAS No. 10049-21-5), disodium hydrogen phosphate (CAS No. 7558-79-4), ammonium sulfate (CAS No. 7783-20-2) and Lysogeny broth (LB) (CAS No. 69-52-3) were purchased from Thermo Fisher Scientific. Antifoam 204 (A6426), IPTG (CAS No. 367-93-1), glucose (CAS No. 50-99-7), guanidine hydrochloride (CAS No. 50-01-1), and imidazole (CAS No. 288-32-4) were obtained from Sigma-Aldrich.

### RHCC-NT expression and purification

The inoculum was grown in Lysogeny broth (LB) containing 100 mg/L ampicillin as selective pressure. Two 2.5 L shake flasks each containing 500 mL of LB medium were inoculated from *E. coli* BL21 (DE3) freshly transformed with pET-15b.his6-TCS-RHCC plasmid and incubated at 37 °C, 220 rpm. Once the OD600 value of inoculum was 30 (after 10 hrs) the bioreactor was inoculated with 500 mL of inoculum (5% of the initial working volume). High density *E. coli* growth in the 15 L New Brunswick BioFlo 115 (Eppendorf) was achieved using a fed-batch fermentation method. Ten liters (working volume) of LB containing 100 mg/L ampicillin was used as the initial fermentation medium. Temperature, pH, and dissolved oxygen were kept constant at 37 °C, 7.5, and 30ppm during the experiment, respectively. Antifoam 204 was added only when needed (between 12–18 hrs of fermentation). The inducer IPTG was added to the media after 9 hrs of fermentation (OD600 = 30) at a final concentration of 1 mM. Samples were taken periodically to monitor the cell growth and glucose concentration. Glucose concentrations were measured using a glucose meter (Bioreactor sciences, BRS GM100). Feeding fermentation medium with 20% glucose solution was initiated when the glucose concentration dropped below 2 g/L, which occurred at 6 h of cultivation. Twenty hours from the start of fermentation, 3 L of medium containing cells removed from the bioreactor and replaced with the same volume (3 L) of LB containing 100 mg/L ampicillin. After 28 hrs of cultivation, cells were pelleted for protein purification. RHCC-NT was purified as described previously. Briefly, pelleted cells are suspended in buffer containing 6 M guanidine hydrochloride and lysed via sonication. After centrifugation (Beckman Coulter, Avanti J-26 XPI) to remove cellular debris, the lysate is passed through a cobalt affinity column and washed with resuspension buffer. The RHCC-NT is eluted with buffer containing 200 mM imidazole. The polyhistidine tag is then removed by thrombin cleavage. A cartoon showing the amino acid sequence is presented in Figure S5.

### Fluorescence binding assay

Initial binding screens were performed by titrating known amounts of a single PAH with varying amounts of RHCC-NT. All assays were performed in 20 mM sodium phosphate solution containing 1% ethanol buffered at pH 7 and adjusted to 150 mM ionic strength. All samples were prepared in triplicate to a final volume of 1 mL and were left to incubate overnight at 25 °C to ensure complete equilibration. PAH fluorescence was then measured as a function of RHCC-NT concentration. Steady-state fluorescence spectra were collected using Fluorolog-3 Horiba Jobin Yvon spectrofluorometer (Edison, NJ). Excitation wavelength was set to 275 nm for naphthalene, 300 nm for acenaphthene, 350 nm for anthracene, 334 nm for chrysene and pyrene, 290 nm for acenaphthylene and fluorene. Excitation and emission slits were set to 1 nm band pass resolution. All samples were measured at 20 °C in a 10 × 3 mm^2^ quartz cuvette. The obtained fluorescence data was used to calculate the binding constant *K*_*d*_ assuming that each PAH binds to one of two equal bindings sites in the RHCC-NT, cavity 2 or 3. The fraction of PAH bound and the concentration of unbound RHCC-NT was determined based on the following equation:

1 $$Fraction\,bound=y=\frac{F-{F}_{0}}{{F}_{\infty }-{F}_{0}}=\frac{{B}_{max}[RHC{C}_{free}]}{{K}_{d}+[RHC{C}_{free}]}$$

where *F* is the experimental fluorescence signal of each sample, *F*_∞_ is the fluorescence of the PAH at infinite protein concentration, *F*_0_ is the fluorescence of the free PAH in solution, *B*_*max*_ is a constant and equal to 1. The y values thus obtained were plotted as a function of free RHCC concentrations. The plotted data were then fit to Equation (1) using Sigma Plot 13 (Point Richmond, CA), yielding the binding constant *K*_*d*_ for each PAH bound to RHCC-NT^27^.

### Time-scale binding assay

The time-scale assay was set-up by addition of pyrene to a 35-fold molar excess of RHCC in 20 mM sodium phosphate solution buffered at pH 7 and adjusted to 150 mM ionic strength. Both solutions were equilibrated to 10 °C prior to the assay. Data was collected on a Fluorolog-3 Horiba Jobin Yvon Spectrofluorometer (Edison, NJ). Excitation wavelength was set to 334 nm; emission was monitored at 384 nm; excitation and emission slits were set to 1 nm band pass resolution. Temperature was kept at 10 °C and controlled with a water bath throughout the experiment. Data was acquired every 1 minute for the first 25 minutes, following by 30 minute intervals until fluorescence signal plateaued and equilibrium achieved.

### Structural elucidation of RHCC-NT:PAH complexes

RHCC-NT:Naphthalene was formed by adding a 5-fold molar excess of naphthalene from a 50% (w/v) naphthalene solution in 95% ethanol to 11.2 mg/mL RHCC in 10 mM Tris pH 7.5 I = 154 mM (NaCl). Due to the lower solubility of pyrene in water, RHCC-NT:Pyrene was formed by heating 225 mg pyrene with 1.6 mL of 5.0 mg/mL RHCC in 20 mM Tris pH 8.0 I = 154 mM (NaCl) to 70 °C and incubating for 1 week. After cooling to 20 °C, excess insoluble pyrene was removed by centrifugation and the complex concentrated to 11.0 mg/mL for crystallization. RHCC-NT:Naph crystal setups were performed by mixing 2 μL protein and 2 μL reservoir containing 1.5 M ammonium sulfate and 0.1 M Tris pH 8.5 at 277 K with a 1 mL reservoir. Crystals appeared after 1 week. RHCC-NT:Pyrene crystals were grown in a 0.7 μL protein and 0.7 μL reservoir solution drop over 50 μL reservoir composed of 25% PEG 3350 m 0.1 M Bis-Tris pH 5.5, 200 mM NaCl (Jena Bioscience Crystals JBScreen JCSG +  + 4). Crystals were soaked with 15% glycerol in reservoir solution for 1 min prior to flash freezing in LN2. Data were collected on a Rigaku rotating anode MM-007HF diffractometer with a wavelength of 1.54178 Å in 1° wedges at 100 K. Data were processed with XDS and the CCP4-package. Phases were calculated in Phaser using a polyserine RHCC model based on a previously solved RHCC structure (pdb code 1FE6)^15^. For RHCC-NT:Pyrene the search model was truncated to two of the four chains of the RHCC tetramer. The structures were built manually and refined refined crystallographically using the Coot suite and the Phenix software package. Coordinates and chemical restraints for pyrene were generated using JLigand and eLBOW.

### Molecular dynamics simulations

Molecular dynamics simulations were performed on three RHCC-PAH complexes (RHCC-naphthalene, RHCC-phenanthrene and RHCC-pyrene) using the GROMACS molecular dynamics simulation package^28^ with the Gromos 43a2 force field and an SPC water model. Each complex is representative of a particular ring structure, one ring (naphthalene), two rings (phenanthrene) and three rings (pyrene). In each case, the simulation data were used to compute the standard free energies for transferring the ligand to cavity 2 and cavity 3 of the RHCC-NT from the solvent. For the RHCC-naphthalene complex and the RHCC-pyrene complex, the starting structures for the simulations were the measured X-ray crystal structures 5VKF and 5VH0, respectively. As no structure of phenanthrene bound to the RHCC-NT was available, it was necessary to construct the RHCC-phenanthrene complex by inserting phenanthrene manually into the RHCC-NT cavities in the same position and orientation as the naphthalene and pyrene ligands. The topologies of all three ligands were generated by PRODRG^29^.

The MD simulation protocols and free energy analysis were based closely on those employed by the authors in a previous investigation^9^ of *RHCC*−*NT* and only the principle features are reproduced here. Each RHCC-PAH complex was solvated in a rectangular simulation box with dimensions 5.2 nm × 5.2 nm × 9.8 nm containing 7900 SPC water molecules and a set of charge neutralizing Na^+^ ions (16 for RHCC-naphthalene and 12 for RHCC-phenanthrene and RHCC-pyrene). A minimum distance of 1.2 nm was maintained between the surface of the RHCC-PAH complex and the walls of the simulation box. Particle mesh Ewald was employed to treat electrostatic interactions and the cut-off radius for non-bonded interactions was taken to be 1.0 nm. The initial X-ray diffraction structure was energy minimized using the method of steepest descent until convergence was achieved, typically around 40–50 kJ/mol. Solvation free energies were calculated by performing an independent series of simulations on a single energy-minimized PAH ligand in a periodic box with dimensions identical to those listed above.

The system was subjected to three position-restrained equilibrations at 50 K, 150 K and 300 K each for a period of 20 ps. During the 2 ns production simulation, the position restraints were removed and the temperature and pressure of the system (RHCC-NT/cavity ligand/solvent bath) were held at *T* = 300*K* and *P* = 1*atm* with thermostats and barostats of the Berendsen type and time constants *τ* = 0.1 *ps*.

The absolute standard state free energy for transferring each PAH ligand from the solvent into one of the cavities of RHCC-NT was computed with the method of double-decoupling^30–32^ using the thermodynamic paths in Figure S3. The upper path represents the conversion of a restrained, fully-interacting ligand bound to the RHCC-PAH complex into a restrained gas-phase particle by turning off the non-bonded interactions while simultaneously restricting the translational degrees of freedom of the ligand to the cavity volume with a flat-bottom harmonic well (FBHW)^33^ with functional form *U*_*FBHW*_(*r* < *r*_0_) = 0; *U*_*FBHW*_(*r* > *r*_0_) = *k*(*r* − *r*_0_)^2^ where *k* = 1000 *kJmol*^−1^*nm*^−2^, *r*_0_ = 0.55 *nm*. (No other conformational, angle or dihedral restraints were employed in the current simulations, where the *RHCC* − *NT* cavities behave simply as storage receptacles.) Δ*G*_1_ is the free energy for this conversion. Δ*G*_2_ is the free energy cost of removing the FBHW restraint, which includes a correction for the standard concentration. The lower path in Figure S3 represents the conversion of a single, fully-interacting, unrestrained ligand immersed in solvent into a free gas-phase particle and Δ*G*_3_ = −Δ*G*_*solv*_ is the associated free energy (Δ*G*_*solv*_ is the solvation free energy). The point symmetry group of each PAH ligand would, in principle, require the inclusion of another term in the free energy Δ*G*_*symm*_ = −*RT lnσ*, where *σ* is the rotational symmetry number. For phenanthrene with point group symmetry *C*_2*v*_, Δ*G*_*symm*_ = −*RT ln*4 = −3.5 *kJ mol*^−1^, while for naphthalene and pyrene with symmetry group *D*_2*h*_, Δ*G*_*symm*_ = −*RT ln*8 = −5.2 *kJ mol*^−1^. However, given the absence of body restraints that would artificially restrict the orientation of the PAH ligands, as mentioned above, this term was omitted from the current analysis. The absolute free energy for transferring a PAH ligand to the cavity from the solvent bath, corrected for the standard state, is

2 $${\rm{\Delta }}{G}_{transfer}^{0}=-\,{\rm{\Delta }}{G}_{1}-{\rm{\Delta }}{G}_{2}+{\rm{\Delta }}{G}_{3}$$

The terms Δ*G*_1_ and Δ*G*_3_ were calculated with the method of Multi-Configurational Thermodynamic Integration (MCTI)^30–37^. Linear scaling of the nonbonded parameters was accomplished using a coupling parameter λ which was varied from λ = 0 (ligand interacting) to λ = 1 (ligand non-interacting) while holding the bond lengths and bond angles fixed. The restraint free energy Δ*G*_2_ was calculated analytically^9^ using:

3 $${\rm{\Delta }}{G}_{2}=+RTln({V}_{FBHW}/{V}_{0})$$

where ${V}_{FBHW}={\int }_{0}^{\infty }exp(-{U}_{FBHW}(r)/RT){d}^{3}r=(4/3)\pi {r}_{0}^{3}+4\pi {r}_{0}^{2}{(\pi RT/k)}^{1/2}+2\pi {r}_{0}(\pi RT/k)+{(\pi RT/k)}^{3/2}\cong 0.90\,n{m}^{3}$ is the effective FBHW simulation volume and *V*_0_ is the standard volume *V*_0_ = 1.660 nm^3^ corresponding to a concentration of 1*M* = 1*molecule*/1.660 nm^3^.

The minimization and equilibration protocols were repeated for each λ before generating the 2 ns production runs. Structures were saved every 0.5 ps and the accumulated set of 4000 structures was used to compute *dG*/*dλ* for each λ. The mean value *dG*/*dλ* was computed using the Gromacs g_analyse routine and the errors were calculated by block averaging. Soft-core interactions with a soft-core parameter *α* = 0.1 were included to obtain smooth *dG*/*dλ* curves and to eliminate discontinuities in the non-bonded parameters and ensure convergence in the limit *λ* → 1.

## Supplementary information

Supplement Information

## References

[1] Ohkouchi N, Kawamura K, Kawahata H (1999). Distributions of three- to seven-ring polynuclear aromatic hydrocarbons on the deep sea floor in the central Pacific. Environmental Science & Technology.

[2] Cerniglia CE (1992). Biodegradation of polycyclic hydrocarbons. Biodegradation.

[3] Mahanty B, Pakshirajan K, Dasu V (2011). Understanding the Complexity and Strategic Evolution in PAH Remediation Research. Crit. Rev. Env. Sci. Tec..

[4] White KL (1986). An overview of immunotoxicology and carcinogenic polycyclic aromatic hydrocarbons. Eviron. Carcinogenesis Rev..

[5] Michel C (2013). Seasonal and PAH impact on DNA strand-break levels in gills of transplanted zebra mussels. Ecotoxicol Environ Saf.

[6] Madison BN, Hodson PV, Langlois VS (2015). Diluted bitumen causes deformities and molecular responses indicative of oxidative stress in Japanese medaka embryos. Aquatic Toxicology.

[7] Dupuis, A. & Ucan-Marin, F. A Literature Review on the Aquatic Toxicology of Petroleum Oil: An Overview of Oil Properties and Effects to Aquatic Biota. *Fisheries and Oceans Canada Science Advisory Secretariat, Ottawa, ON* (2014).

[8] Bourgeault A, Gourlay-France C (2013). Monitoring PAH contamination in water: comparison of biological and physico-chemical tools. Sci Total Environ.

[9] McDougall M (2017). Archaea S-layer nanotube from a “black smoker” in complex with cyclo-octasulfur (S-8) rings. Proteins-Structure Function and Bioinformatics.

[10] Kang HJ, Lee SY, Kwon JH (2016). Physico-chemical properties and toxicity of alkylated polycyclic aromatic hydrocarbons. Journal of Hazardous Materials.

[11] Aguiar J, Carpena P, Molina-Bolivar JA, Ruiz CC (2003). On the determination of the critical micelle concentration by the pyrene 1: 3 ratio method. Journal of Colloid and Interface Science.

[12] Kalyanasundaram K, Thomas JK (1977). Environmental effects on Vibronic Band Intensities in Pyrene Monomer Fluorescence and heir Application in Studies of Micellar Systems. Journal of the American Chemical Society.

[13] Dong DC, Winnik MA (1982). The PY Scale of Solvent Polarities- Solvent Effects on the Vibronic Fine-Structure of Pyrene Fluorescence and Empirical Correlations with ET-Value and Y-Value. Photochemistry and Photobiology.

[14] Dong DC, Winnik MA (1984). The PY Scale of Solvent Polarities. Canadian Journal of Chemistry-Revue Canadienne De Chimie.

[15] Stetefeld J (2000). Crystal structure of a naturally occurring parallel right-handed coiled coil tetramer. Nat Struct Biol.

[16] Rizzo RC, Aynechi T, Case DA, Kuntz ID (2006). Estimation of absolute free energies of hydration using continuum methods: Accuracy of partial, charge models and optimization of nonpolar contributions. Journal of Chemical Theory and Computation.

[17] Charlier L, Nespoulous C, Fiorucci S, Antonczak S, Golebiowski J (2007). Binding free energy prediction in strongly hydrophobic biomolecular systems. Phys Chem Chem Phys.

[18] Fujitani H (2005). Direct calculation of the binding free energies of FKBP ligands. J Chem Phys.

[19] Burkhard P, Stetefeld J, Strelkov SV (2001). Coiled coils: a highly versatile protein folding motif. Trends Cell Biol.

[20] McFarlane AA, Orriss GL, Stetefeld J (2009). The use of coiled-coil proteins in drug delivery systems. Eur J Pharmacol.

[21] Ozbek S, Engel J, Stetefeld J (2002). Storage function of cartilage oligomeric matrix protein: the crystal structure of the coiled-coil domain in complex with vitamin D(3). EMBO J.

[22] McFarlane A (2012). The pentameric channel of COMPcc in complex with different fatty acids. PloS one.

[23] McDougall M (2018). Reductive power of the archaea right-handed coiled coil nanotube (RHCC-NT) and incorporation of mercury clusters inside protein cages. J Struct Biol.

[24] Zegar IS (1996). Adduction of the human N-ras codon 61 sequence with (-)-(7S, 8R, 9R, 10S)-7,8-dihydroxy-9,10-epoxy-7,8,9,10-tetrahydrobenzo[a] pyrene: structural refinement of the intercalated SRSR(61,2) (-)-(7S, 8R, 9S, 10R)-N6-[10-(7,8,9,10- tetrahydrobenzo[a]pyrenyl)]−2’-deoxyadenosyl adduct from 1H NMR. Biochemistry.

[25] Sansen S (2007). Adaptations for the oxidation of polycyclic aromatic hydrocarbons exhibited by the structure of human P450 1A2. J Biol Chem.

[26] Bamforth SM, Singleton I (2005). Bioremediation of plycyclic aromatic hydrocarbons: current knowledge and future directions. Journal of Chemical Technology and Biotechnology.

[27] Rossi AM, Taylor CW (2011). Analysis of protein-ligand interactions by fluorescence polarization. Nat Protoc.

[28] Van der Spoel D (2005). GROMACS: Fast, flexible, and free. Journal of Computational Chemistry.

[29] Schuttelkopf AW, van Aalten DMF (2004). PRODRG: a tool for high-throughput crystallography of protein-ligand complexes. Acta Crystallographica Section D-Biological Crystallography.

[30] Gilson MK, Given JA, Bush BL, McCammon JA (1997). The statistical-thermodynamic basis for computation of binding affinities: A critical review. Biophysical Journal.

[31] Hamelberg D, McCammon JA (2004). Standard free energy of releasing a localized water molecule from the binding pockets of proteins: Double-decoupling method. Journal of the American Chemical Society.

[32] Deng Y, Roux B (2006). Calculation of standard binding free energies: Aromatic molecules in the T4 lysozyme L99A mutant. Journal of Chemical Theory and Computation.

[33] Helms, V. & Wade, R. C. Hydration energy landscape of the active site cavity in cytochrome P450cam. *Proteins-Structure Function and Genetics* **32**, 381–396, doi:10.1002/(sici)1097-0134(19980815)32:3<381::Aid-prot12>3.0.Co;2-5 (1998). 9715913

[34] Helms V, Wade RC (1998). Computational alchemy to calculate absolute protein-ligand binding free energy. Journal of the American Chemical Society.

[35] Straatsma, T. P. & McCammon, J. A. In *Methods in Enzymology* Vol. 202 497–511, (Academic Press, 1991). 10.1016/0076-6879(91)02025-5 1784186

[36] Boresch S, Karplus M (1995). The Meaning of Component Analysis - Decomposition of the Free-Energy in Terms of Specific Interactions. Journal of Molecular Biology.

[37] Hermans J, Shankar S (1986). The Free-Energy of Xenon binding to Myoglobin from Molecular-Dynamics Simulations. Israel Journal of Chemistry.

