# Supplementary material for: Proteinaceous Nano container Encapsulate Polycyclic Aromatic Hydrocarbons
Source: Sci Rep. 2019 Jan 31;9:1058. doi: 10.1038/s41598-018-37323-x (PMC6355809; doi:10.1038/s41598-018-37323-x)
Supplement: Supplementary file 1 — Supplement Information [file 41598_2018_37323_MOESM1_ESM.docx]

**SUPPLEMENTAL INFORMATION**

**Proteinaceous Nano container Encapsulate Polycyclic Aromatic Hydrocarbons**

Matthew McDougall^1,2^, Olga Francisco^1,2^, Candice Harder-Viddal^3^ , Roy Roshko^4^, Fabian Heide^1^, Shubleen Sidhu^1^, Mazdak Khajehpour^1^, Jennifer Leslie^5^, Vince Palace^6^, Gregg T Tomy^1,2^ and Jörg Stetefeld^1,2*^

^1^ Department of Chemistry, University of Manitoba, 144 Dysart Road, Winnipeg, Manitoba, R3T 2N2 Canada

^2^ Centre for Oil and Gas Research and Development (COGRAD), 144 Dysart Road, Winnipeg, MB, R3T 2N2 Canada

^3^ Department of Chemistry and Physics, Canadian Mennonite University, 500 Shaftesbury Blvd, Winnipeg, MB, R3T 2N2 Canada

^4^ Department of Physics and Astronomy, University of Manitoba, 30A Sifton Rd, Winnipeg, MB, R3T 2N2 Canada

^5^ Stantec Consulting Inc., 500-311 Portage Ave., Winnipeg, MB, R3B 2B9 Canada

^6^ IISD-Experimental Lakes Area, 111 Lombard Ave, Winnipeg MB R3B 0T4 Canada

**Figure S1.** Fluorescence emission of 61.5µM RHCC-NT (λ_exc_=275 nm). Slits were set to 1 nm.

**Figure S2.** Change in fluorescence intensity as a function of RHCC concentration in the presence of phenanthrene (left) and benz[a]anthracene (right). Both compounds exhibit no binding to RHCC-NT.

$$\left[\left(L+U_{FBHW}\right)_{RHCC}\right]_{sol}\to^{\Delta G_{1}} \left[\left(L_{gas}+U_{FBHW}\right)_{RHCC}\right]_{sol}\to^{\Delta G_{2}} \left[\left(L_{gas}+U_{FBHW}^{0}\right)_{RHCC}\right]_{sol}$$

$$\left[L\right]_{sol}\to^{\Delta G_{3}} \left[L_{gas}\right]_{sol}$$

**Figure S3**. A schematic diagram showing the two thermodynamic paths used to calculate the absolute free energy for the transfer of $S_{8}$ from the solvent bath to cavity 2 of RHCC tetrabrachion $\left( RHCC \right)$. The rectangle represents the FBHW restraint. The standard state transfer free energy from solvent to protein is $\Delta G_{transfer}^{0}=- \Delta G_{1}- \Delta G_{2}+ \Delta G_{3}$.

**Figure S4.** Electron density of the bound PAH. 2FoFc electron density maps contoured at 2σ of naphthalene in cavity 2 (A) and cavity 3 (B), and at 1.5σ of pyrene in cavity 2 (C) and 3 (D). The RHCC-NT is displayed in cartoon representation with sidechains essential for cavity formation shown as sticks. The foremost chain is omitted for clarity.

GSIINETADDIVYRLTVIIDDRYESLKNLITLRADRLEMIINDNVSTILASI

**Figure S5.** Sequence of the RHCC-NT used in the experiments.
